# Correlation between Doppler Echocardiography and Right Heart Catheterization Assessment of Systolic Pulmonary Artery Pressure in Patients with Mitral Regurgitation: A Prospective Observational Study

**DOI:** 10.31083/j.rcm2307245

**Published:** 2022-06-28

**Authors:** Ali Haddad, Olga Tsarenko, Cynthia Szalai, Ahmed Mohamed, Marcel Hochreiter, Marc Moritz Berger, Bastian Schmack, Arjang Ruhparwar, Thorsten Brenner, Sharaf-Eldin Shehada

**Affiliations:** ^1^Department of Anesthesiology and Intensive Care Medicine, University Hospital Essen, University Duisburg-Essen, 45147 Essen, Germany; ^2^Department of Thoracic and Cardiovascular Surgery, West German Heart and Vascular Canter, University Hospital Essen, University Duisburg-Essen, 45141 Essen, Germany

**Keywords:** Doppler echocardiography, right-side heart catheterization, pulmonary artery catheter, mitral valve regurgitation

## Abstract

**Background:**

Pulmonary hypertension (PH) is common in patients with left-side valvular diseases, especially with mitral regurgitation (MR). Measurement using pulmonal artery catheter (PAC) is the gold standard to asses pulmonary vascular pressures. During mitral valve surgery echocardiography is routinely used for valvular management and to evaluate pulmonary hemodynamic. The accuracy of echocardiographic measurements is controversial in the literature. We aimed to evaluate the reliability and accuracy of the noninvasive measurement for systolic pulmonary artery pressure (SPAP) using Doppler echocardiography compared to the invasive measurement using PAC in patients presenting with MR undergoing surgery.

**Methods:**

This prospective observational study evaluated 146 patients with MR undergoing cardiac surgery between 09/2020 and 10/2021. All patients underwent simultaneous SPAP assessment by PAC and transesophageal echocardiography at three different time points: before heart-lung-machine (HLM), after weaning from HLM and at the end of surgery.

**Results:**

Mean patients’ age was 61 ± 11.5 years, and 51 (35%) patients were female. Most of patients presented with severe MR (n = 126; 86.3%) or endocarditis (n = 18; 12.3%). Patients underwent either isolated mitral valve surgery (n = 65; 44.5%) or mitral valve surgery combined with other surgeries (n = 81; 55.5%). Mean SPAP was underestimated by transesophageal echocardiographic measurement in comparison to PAC measurement before HLM (41.9 ± 13.1 mmHg vs. 44.8 ± 13.8 mmHg, *p *< 0.001), after weaning from HLM (37.6 ± 9.3 mmHg vs. 42.4 ± 10.1 mmHg, *p *< 0.001), and at the end of surgery (35.6 ± 9.1 mmHg vs. 39.9 ± 9.9 mmHg, *p *< 0.001). This difference remained within the sub-analysis in patients presented with moderate or severe PH during all the time points. Bland-Altman analysis showed that transesophageal echocardiographic measurement underestimate SPAP in comparison to PAC as these two approaches are significantly different from one another.

**Conclusions:**

In patients presented with MR, transesophageal Doppler echocardiography could asses the presence of PH with high probability. This assessment is however underestimated and the use of PAC in those patients to diagnose, classify and monitor the therapy of PH remains recommended if required.

## 1. Introduction

Mitral valve regurgitation (MR) is one of the most frequent heart valve diseases worldwide [1]. Pulmonary hypertension (PH) is a common pathology in patients with left-side valvular diseases [2]. Many studies report increased mortality in patients presenting with PH undergoing mitral valve surgery and considered it to be a marker for a poor outcome after surgery [3, 4]. The assessment of PH is essential for risk stratification, and is one of the components of the EuroSCOREs [5, 6]. According to European-Society of Cardiology/European-Respiratory-Society (ESC/ERS) guidelines, the right heart catheterization (RHC) using pulmonary artery catheter (PAC) is the gold standard for direct measurement of pulmonary artery pressure (PAP) [7]. Accordingly, PH is defined by a mean PAP ≥25 mmHg [7]. Based on the EuroSCORE II, moderate PH is defined as a systolic pulmonary artery pressure (SPAP) of ≥31 mmHg, and severe PH as SPAP of ≥55 mmHg [6]. The RHC is an invasive tool and potentially associated with severe complications. Transesophageal echocardiography (TEE) is a useful routine clinical tool during cardiac surgery. The noninvasive estimation of SPAP by Doppler echocardiography is widely used in clinical routine [7].

SPAP assessment using echocardiography has been described since more than three decades [8, 9, 10]. For calculation of SPAP, the right ventricular systolic pressure (RVSP) measured by maximal flow velocity of the tricuspid valve regurgitation (TR) is added to the right atrial pressure (RAP) measured by central venous catheter (CVC) [7, 11, 12]. Ever since, Doppler echocardiography is routinely used to estimate PH within the daily practice. So far, there are just few studies with small sample size which investigated the correlation between SPAP measurement by Doppler echocardiography and invasive measurement using a PAC [13, 14, 15, 16, 17, 18, 19, 20]. In some of these studies, the accuracy of echocardiographic SPAP estimation has been questioned [13, 14, 15, 16, 17, 18, 19, 20]. Furthermore, to our knowledge the correlation between invasive and noninvasive estimation of pulmonary artery pressure simultaneously in patients with mitral valve regurgitation has not been investigated yet.

Therefore, this study was performed in order to investigate the correlation between simultaneous noninvasive transesophageal Doppler echocardiography and invasive right heart catheter estimation of SPAP in a prospective cohort of patients presenting with MR undergoing cardiac surgery.

## 2. Material and Methods

### 2.1 Patient Population and Study Design

**Fig. 1.**
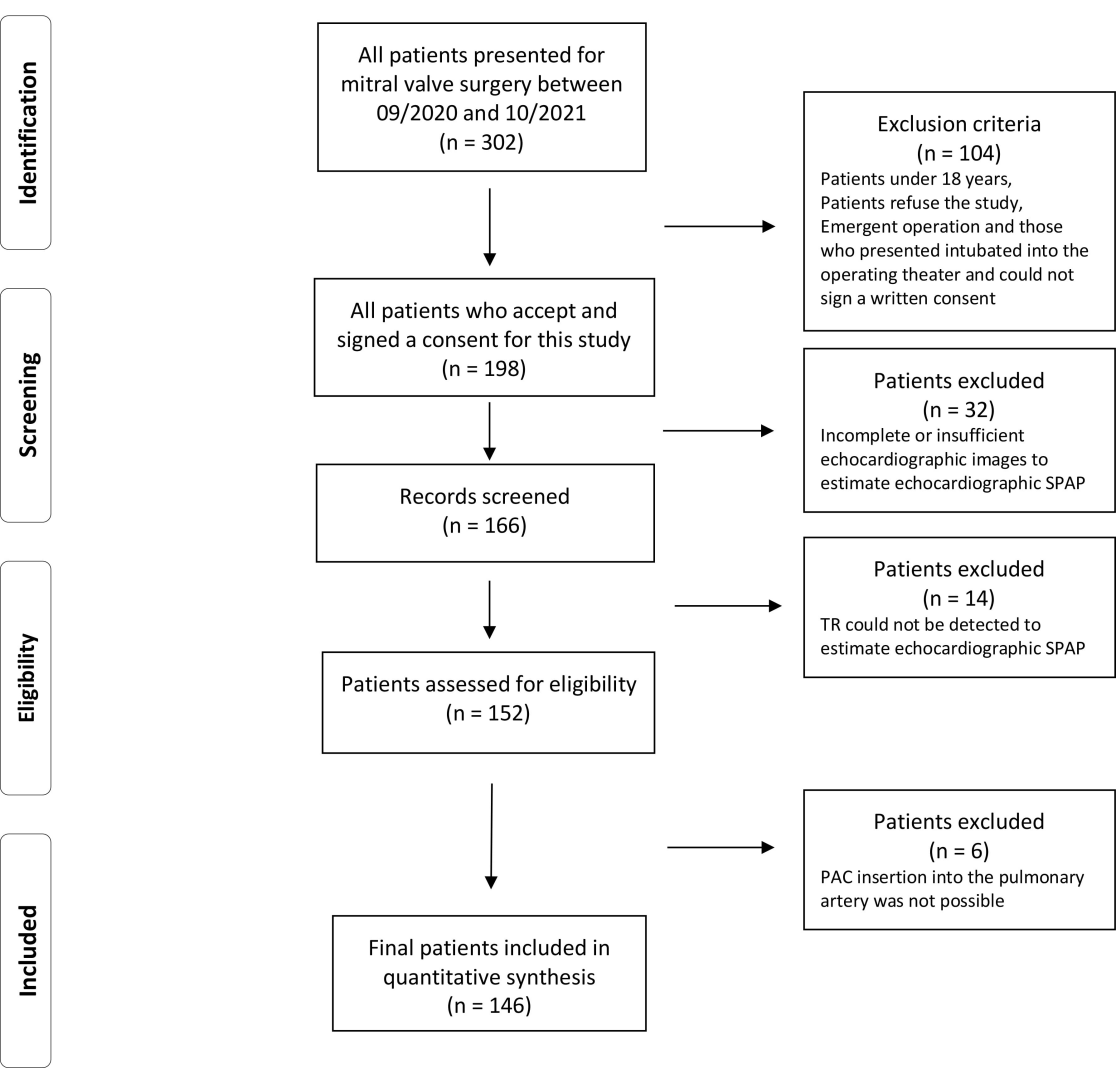
**Study Flowchart**.

The study obtained a review board approval according to the University-Hospital-Ethics-Committee (Ref# 20-9403-BO). The study is a single-center prospective observational one included patients presenting with MR undergoing mitral valve repair or replacement at the University Hospital Essen over a one-year period between 09/2020 and 10/2021. Exclusion criteria were: patients <18 years, those who refused to participate in the study, patients presenting for emergency surgery, and those who could not sign a written consent. In total, 198 patients were primarily included and recorded in our study database. Thereafter, data were screened for eligibility, extracted and then evaluated. However, records of 32 patients did not have a sufficient transesophageal echocardiographic image quality to estimate SPAP, records in another 14 patients did not show TR necessary to estimate SPAP via echocardiography, and in 6 patients PAC insertion into the pulmonary artery was not possible. Finally, data from 146 patients with simultaneous hemodynamic assessment by PAC and transesophageal echocardiography were included in this study as shown within the study flowchart (Fig. 1).

### 2.2 Assessment of Pulmonary Artery Pressure 

After induction of general anesthesia and endotracheal intubation, all 146 patients received central venous catheter and a pulmonary artery catheter via a 8.5 French sheath introducer through a central vein. The SPAP values were measured simultaneously by PAC and transesophageal echocardiography at three different time points: first, after induction of anesthesia and before heart-lung-machine, second, after weaning from the HLM, and finally at the end of surgery and prior transfer to the ICU. In 18 patients, who received concomitant tricuspid valve reconstruction, the PAC was pulled back from the catheter sheath to the central vein after the first measurement and could not be re-introduced to the pulmonary artery after valve repair. Additionally, the echocardiographic evaluation of SPAP could not be done in these 18 patients post valve repair. This in turn allowed a total of 402 measurements of SPAP for all three modalities. PAP measurements were carried out by two different experienced cardiothoracic anesthesiologists of which one was responsible for the transesophageal echocardiography measurements and the other one for the PAC measurements. Both investigators were blinded to the measurements made by the other investigator.

### 2.3 Pulmonary Artery Catheter 

All patients underwent right-side heart catheterization with a PAC. PAC was used to report hemodynamic values including: pulmonary artery systolic and diastolic pressures (SPAP & DPAP), right atrial pressure (RAP), pulmonary capillary wedge pressure (PCWP), systemic and pulmonary vascular resistance (SVR & PVR). Mean PAP was calculated with the equation [DPAP + 1/3(SPAP-DPAP)] [21]. Cardiac output (CO) was determined using the thermodilution technique [21]. Stroke volume (SV) was calculated as CO divided by heart rate (HR) [CO/HR]. Indexes of CO, SV, SVR and PVR variables were calculated via dividing each value with the body surface area (BSA) yielding cardiac index (CI), stroke volume index (SVI), systemic vascular resistance index (SVRI) and pulmonary vascular resistance index (PVRI).

### 2.4 Transesophageal Echocardiography 

Standardized transesophageal echocardiography (TEE) examination was performed in all cases in our institution by experienced cardiothoracic anesthesiologist who was certified by the National Board of Echocardiography. The TEE examination included assessment of all heart valves, and the left ventricular ejection fraction (LVEF) by the Simpson method. Basically, right ventricular systolic pressure (RVSP) represents the systolic pulmonary artery pressure in absence of pulmonary valve pathology [8]. The echocardiographic RVSP was calculated by adding the trans-tricuspid pressure gradient (TPG) to the measured RAP as represented by the CVP. TPG was calculated by the modified Bernoulli equation, which was drawn by the peak systolic velocity flow across the regurgitating tricuspid valve with the continuous wave Doppler (TPG = 4 X Vmax2) [9]. The modified Bernoullie equation is agnostic to the direction of the blood flow; it merely measures the pressure gradient across a small orifice, the flow through this orifice will depend on the pressure gradient across it.

### 2.5 Statistical Analysis 

Statistical analysis was performed using the SPSS-software (version 27.0. IBM Crop., Armonk, NY, USA). Continuous data were expressed as means and standard deviation (SD) or medians with the 25th–75th interquartile ranges (IQR), as appropriate, and categorical data were expressed as percentages and frequencies. Differences between the two types of measurements were compared by *t*-test. All reported *p* values are two-sided and a value of *p *< 0.05 was considered statistically significant. Agreement of measurements was assessed by way of Bland–Altman plots [22]. Finally, Excel 2016 software (version 16.0, Microsoft, Albuquerque, NM, USA) was used to create clustered bars, which show the difference in both approaches in a diagram.

## 3. Results

### 3.1 Preoperative Data

The preoperative patient characteristics are described in Table 1. Mean patients’ age was 61 ± 11.5 years, and 51 (35%) patients were male. More than half of the patients (81, 55.5%) presented with impaired functional capacity (class III or IV) according to the New York Heart Association (NYHA) functional classification and 15 patients had prior cardiac surgery. Most of the patients (145, 99.3%) presented with moderate to severe MR, 18 patients had active endocarditis, and 18 patients presented with concomitant severe TR. Moderate to severe PH was diagnosed in 114 (78.1%) patients. Additionally, 33 (22.6%) patients presented with concomitant severe aortic valve pathology, another 33 (22.6%) patients had severe coronary artery disease and 10 (6.9%) patients had patent foramen ovale.

**Table 1.** **Baseline characteristics**.

Variable		Patients (n = 146)
Demographics		
	Age, years	61 ± 11.5
	Gender, male	95 (65)
	BMI*, kg/$m^{2}$	25.8 ± 4.3
Risk factors & comorbidities		
	Arterial hypertension	106 (72.6)
	Diabetes mellitus	36 (24.7)
	COPD*	12 (8.2)
	Peripheral vascular disease	3 (2.1)
	Cerebrovascular disease	14 (9.6)
	Preoperative creatinine level, mg/dL	1.1 ± 0.7
	Preoperative impaired kidney function	14 (9.6)
	Atrial fibrillation	34 (23.3)
	Anticoagulation (OAK*s or NOAK*s)	47 (32.2)
	NYHA* III-IV	81 (55.5)
	Prior cardiac surgery	15 (10.3)
Mitral valve pathology		
	Mild regurgitation	1 (0.7)
	Moderate regurgitation	19 (13.0)
	Severe regurgitation	126 (86.3)
	Endocarditis	18 (12.3)
Tricuspid valve pathology		
	Mild regurgitation	78 (53.4)
	Moderate regurgitation	50 (34.2)
	Severe regurgitation	18 (12.3)
	Other cardiac pathologies	
	Severe aortic valve pathology	33 (22.6)
	Severe coronary artery disease	33 (22.6)
	Patent foramen ovale	10 (6.9)
	Presence and severity of pulmonary hypertension	
	None (SPAP* 0–30 mmHg)	32 (21.9)
	Moderate (SPAP 31–55 mmHg)	87 (59.6)
	Severe (SPAP >55 mmHg)	27 (18.5)
Operation risk scores		
	Logistic EuroSCORE*	3.3 (2–7.5)
	EuroSCORE II	1.7 (0.8–2.6)
	STSROM*	0.7 (0.4–1.8)
	STSROMM*	7.3 (5.1–13)

Data are presented as mean ± SD, number (%) or median (interquartile range). BMI, Body mass index; COPD, Chronic obstructive pulmonary disease; NYHA, New York Heart Association functional classification; SPAP, Systolic pulmonary artery pressure; EuroSCORE, European System for Cardiac Operative Risk Evaluation. STSROM/M, Society of Thoracic Surgery Risk of Mortality and/or Morbidity.

### 3.2 Echocardiographic and Hemodynamic Data

Table 2 summarizes echocardiographic characters and the hemodynamic data prior to HLM. Mean left ventricular ejection function was 54 ± 10%, and 32 (21.9%) patients presented with impaired LVEF (<50%). The mean effective regurgitation orifice area of the diseased mitral valves was 0.6 ± 0.2 ${\mathrm{cm}}^{2}$ and the mean size of the vena contracta was 7.1 ± 1.2 mm. PAC was used to evaluate the hemodynamics prior to HLM; mean SPAS was 44.8 ± 13.8 mmHg, mean CVP was 11.9 ± 7.2 mmHg, mean cardiac output was 3.5 ± 1.2 L/min, median wedge pressure was 14 mmHg, and the mean systemic vascular resistance index (SVRI) was 3109.2 ± 1223.3 (${\mathrm{WU.m}}^{2}$), and pulmonary vascular resistance index (PVRI) was 557.5 ± 341.1 (${\mathrm{WU.m}}^{2}$).

**Table 2.** **Preoperative echocardiographic and hemodynamic data**.

Variable		Patients (n = 146)
Echocardiographic data		
	E/A ratio	2.4 ± 1.1
	Deceleration time, ms	235.8 ± 112.5
	E´ septal, cm/s	7.5 ± 2.7
	E´ lateral, cm/s	8.6 ± 3.1
	E/E´ ratio	13.6 ± 7.0
	Vena contracta, mm	7.1 ± 1.2
	EROA*, ${\mathrm{cm}}^{2}$	0.6 ± 0.2
	Mean left ventricular ejection fraction, (%)	54 ± 10
	Impaired left ventricular function (LVEF* <50%)	32 (21.9)
	sPAP*, mmHg	41.9 ± 13.1
Hemodynamic data using PAC*		
	sPAP*, mmHg	44.8 ± 13.8
	dPAP, mmHg	20.1 ± 7.9
	mPAP, mmHg	29.3 ± 9.7
	CVP*, mmHg	11.9 ± 7.2
	Wedge pressure, mmHg	14 (9–17)
	Heart rate, beat/min	62 ± 15
	Cardiac output, L/min	3.5 ± 1.2
	Cardiac index, L/min/$m^{2}$	1.7 ± 0.5
	SVRI*, ${\mathrm{WU.m}}^{2}$	3109.2 ± 1223.3
	PVRI*, ${\mathrm{WU.m}}^{2}$	557.5 ± 341.1

Data are presented as mean ± SD or median (interquartile range). EROA, Effective regurgitation orifice area; PAC, Pulmonary artery catheter; sPAP, Systolic pulmonary artery pressure; dPAP, diastolic pulmonary artery pressure; mPAP, Mean pulmonary artery pressure; CVP, Central venous pressure; SVRI, Systemic vascular resistance index; PVRI, Pulmonary vascular resistance index.

### 3.3 Correlation between Noninvasive and Invasive Estimation of SPAP

**Fig. 2.**
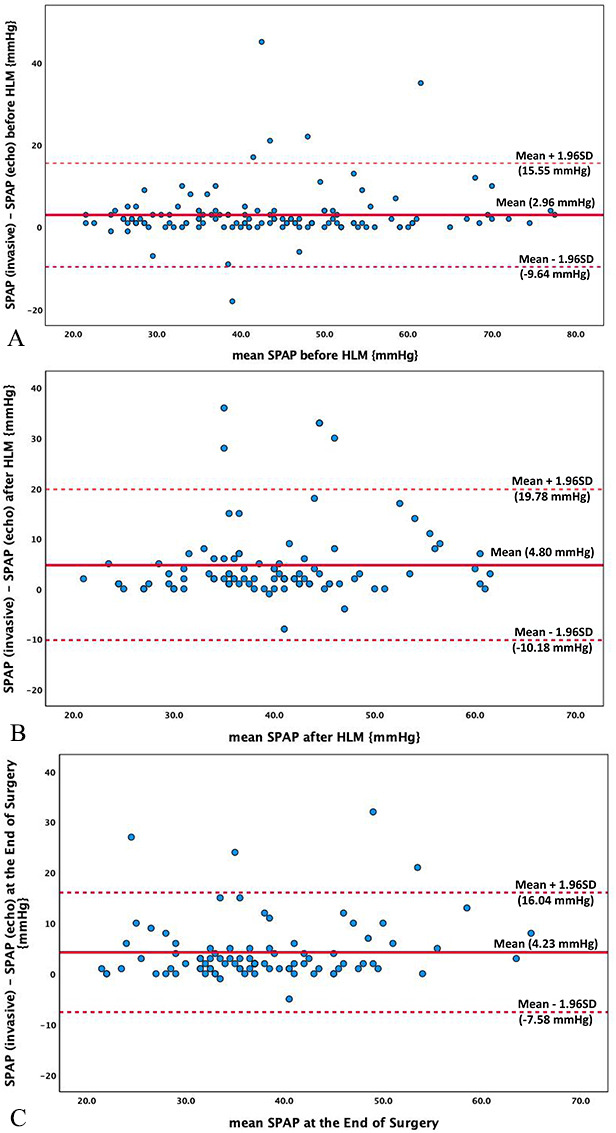
**Bland-altman plots assessing the correlation between systolic pulmonary artery pressure measured either invasively by pulmonary artery catheter or noninvasively by doppler echocardiography**. (A) Before HLM. (B) After HLM. (C) At the end of surgery.

**Fig. 3.**
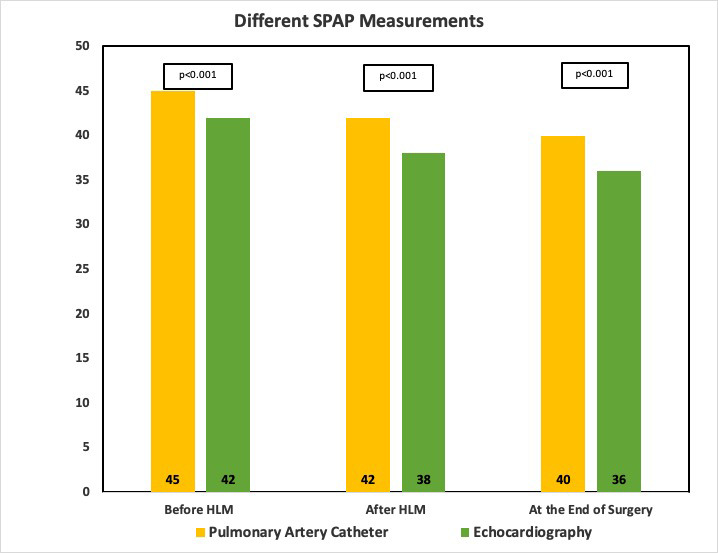
**Clustered bars showing different SPAP measurements using pulmonary artery catheter and Doppler echocardiography**.

Table 3 summarizes correlation between noninvasive and invasive estimation of SPAP. Mean SPAP showed a significant underestimation of echocardiographic measurements in comparison to PAC measurements before HLM (41.9 ± 13.1 vs. 44.8 ± 13.8, *p *< 0.001), after weaning from HLM (37.6 ± 9.3 vs. 42.4 ± 10.1, *p *< 0.001), and at the end of surgery (35.6 ± 9.1 vs. 39.9 ± 9.9, *p *< 0.001). This difference remained in the sub-analysis in patients presented with moderate or severe PH during all the three time points of assessment as reported in Table 4. Bland-Altman analysis showed that these two approaches are significantly different from one another (Fig. 2A,B,C). Finally, Fig. 3 illustrate the difference between both measurements in diagrammatic clustered bars.

**Table 3.** **Differences between PAP measurement using echocardiographic and PAC**.

Severity	Time of measurement	SPAP* with PAC	SPAP* with TEE	*p*-value
Mean value for all patients, mmHg				
	Before HLM*	44.8 ± 13.8	41.9 ± 13.1	<0.001
	After weaning from HLM	42.4 ± 10.1	37.6 ± 9.3	<0.001
	At the end of surgery	39.9 ± 9.9	35.6 ± 9.1	<0.001
No PAH* (sPAP* 0–30 mmHg)				
	Before HLM	27.2 ± 2.2	26.7 ± 5.3	0.598
	After weaning from HLM	35.1 ± 7.8	32.1 ± 6.8	0.003
	At the end of surgery	35 ± 8.7	31.6 ± 8.2	0.001
Moderate PAH (sPAP* 31–55 mmHg)				
	Before HLM	43.3 ± 7	40.9 ± 7.6	<0.001
	After weaning from HLM	44.2 ± 9.8	38.5 ± 9.3	<0.001
	At the end of surgery	40.7 ± 9.6	35.9 ± 8.6	<0.001
Severe PAH (sPAP* >55 mmHg)				
	Before HLM	66.1 ± 7.5	59.1 ± 12.4	0.004
	After weaning from HLM	45.7 ± 10	42.1 ± 9.3	0.001
	At the end of surgery	44.5 ± 10.3	41.2 ± 9.6	0.006

Data are presented as mean ± SD. PAH, Pulmonary arterial hypertension; HLM, Heart-lung-machine; SPAP, Systolic pulmonary artery pressure; PAC, Pulmonary artery catheter; TEE, Transesophageal echocardiography.

**Table 4.** **Operative and early postoperative outcomes**.

Variable		Patients (n = 146)
Indication for surgery		
	Elective	128 (87.7)
	Urgent (endocarditis)	18 (12.3)
Surgical outcomes		
	Minimal invasive	28 (19.2)
	Conventional procedure	118 (80.8)
	Mitral valve repair	120 (82.2)
	Mitral valve replacement	26 (17.8)
	Isolated mitral valve surgery	65 (44.5)
	Combined mitral valve surgery	81 (55.5)
	Combined with aortic valve replacement	33 (22.6)
	Combined with tricuspid valve repair	18 (12.3)
	Combined with CABG*	33 (22.6)
	PFO* closure	10 (6.8)
	More than two procedures	39 (26.7)
Intraoperative use of NO* or Iloprost®		
	Only Iloprost®	39 (26.7)
	NO* and Iloprost®	10 (6.9)
Postoperative outcomes		
	ICU*- stay, days	2 (2–6.5)
	Hospital- stay, days	12 ± 5.8
	30-day mortality	14 (9.6)

Data are presented as mean ± SD, number (%) or median (interquartile range). CABG, Coronary artery bypass grafting; PFO, Patent foramen ovale; NO, Nitrous Oxide; ICU, Intensive care unit.

### 3.4 Operative and Postoperative Outcomes

Table 4 reports perioperative outcomes. Patients presented with active endocarditis underwent urgent surgery 18 (12.3%). Minimal invasive surgery was performed in 28 (19.2%) patients. Most of the patients 120 (82.2%) underwent mitral valve repair. More than half of the patients 81 (55.5%) underwent concomitant procedure: tricuspid valve repair in 18 (12.3%) patients, aortic valve replacement in 33 (22.6%) and coronary artery bypass grafting in 33 (22.6%) and PFO closure in 10 (6.9%) patients. Of these, 39 (26.7%) patients underwent more than two procedures. Patient with severe PH received intraoperative prostacyclin analogues (Iloprost®) alone in 39 (26.7%) or combined with nitrous oxide in 10 (6.9%) patients. Finally, median ICU-stay was two days, and 30-day mortality was reported in 14 (9.6%) patients.

## 4. Discussion

So far, only few studies have been performed to evaluate the correlation between simultaneous noninvasive estimation of SPAP by transesophageal Doppler echocardiography and invasive measurement of SPAP via right-side heart catheterization. Most of these studies have investigated nonhomogeneous groups of patients that presented with different cardiac pathologies, which in turn might impact outcomes. Therefore, we decided to perform a prospective study to analyze this correlation in a cohort of patients presenting with MR undergoing surgery, where SPAP was measured simultaneously using Doppler echocardiography and PAC from two different experienced cardiothoracic anesthesiologists, additionally SPAP measurements were done at three different time points perioperatively.

In 146 patients undergoing mitral valve surgery due to mitral regurgitation, SPAP has been measured 402 times with each modality via PAC and Doppler echocardiography simultaneously before and after HLM, and at the end of surgery. The main findings in our study are: (1) Doppler echocardiography is a routinely used, noninvasive feasible tool to screen patients with pulmonary hypertension. (2) Echocardiography in patients with mitral valve regurgitation underestimates the SPAP in comparison to right-side heart catheterization. The reported difference is significant between both modalities, regardless the presence of PH. (3) Bland-Altman analysis proved that measurement by echocardiography underestimate the measurement made by PAC as these two measurements are significantly different from each another and cannot provide a useful level of agreement.

Earlier studies have reported that the invasive measurement of pulmonary artery pressure using right-side heart catheterization via PAC to be the gold standard manner for the diagnosis of PH [7, 8, 9, 10, 11]. This approach is associated with an in-hospital mortality of 0,0055% [12]. Cost-beneficially, it is not practical to insert a PAC in all patients presented for cardiac surgery. Echocardiography is, however, a routine and fundamental in all patients undergoing cardiac surgery, it is frequently used to screen and monitor heart valves and both ventricular function. Based on its non-invasive nature, wide availability and cost effectiveness in comparison to PAC, it could be also used to diagnose and monitor the therapy of severe PH and control its progression over time [7, 11, 23].

To the best of our knowledge, the reliability of Doppler echocardiography to estimate SPAP noninvasively has been assessed in small retrospective studies with controversially results. D’Alto *et al*. [16] evaluated 161 patients with suspected PH. They reported that echocardiography allows for accurate measurement of PH, however, with moderate precision [16]. In a cohort of 374 lung transplant candidates, 52% of pressure estimations by echocardiography were reported to be inaccurate with more than 10 mmHg difference compared to the measured pressure using PAC [15]. Rich *et al*. [14] reported in 160 patients with PH a moderate correlation (r = 0.68), where Doppler echocardiography estimation of SPAP were determined to be inaccurate in 50.6% of patients despite sort of simultaneous measurements. Fischer *et al*. [13] evaluated the accuracy of Doppler echocardiography for estimating pulmonary artery pressure and cardiac output in 65 patients within one hour after they received a PAC. Doppler echocardiography was reported inaccurate (defined as being >10 mmHg of the invasive measurement) in 48% of cases. On the other hand, several studies showed a good correlation between the two modalities. For instance, Amsallem *et al*. [19] examined a population with PH or advanced lung disease and reported a correlation of r = 0.84 and an accuracy of 72% of the Doppler echocardiography. More recently, Schewel *et al*. [18] reported in their retrospective analyzes of 1400 patients with aortic stenosis a very good correlation between SPAP measurement via PAC and echocardiography performed within five-day interval. Notably, a cut-off value of RVSP >34 mmHg is highly associated with PH [7] according to the ESC/ERS guidelines and a further evaluation of symptomatic patients is recommended if RVSP >40 mmHg according to the ACCF/AHA guidelines [23]. Greiner *et al*. [20] reported in one of largest cohorts including 1695 cardiac patients that an echocardiographic SPAP cutoff of ≥36 mmHg has the highest sensitivity (87%) and specificity (79.1%) for PH diagnosis (invasive MPAP ≥25 mmHg).

In the current prospective observational study, we evaluated the difference between both measurement of pulmonary artery pressure in 146 patients presented with MR. PH is known to be a common pathology in patients with left-side valvular diseases [2]. The majority of patients (n = 120; 82.2%) underwent mitral valve repair with different repair techniques [24]. Valve repair was also possible even in patients presented with valve endocarditis, repair techniques in cases of endocarditis was earlier reported [25]. Mitral valve replacement was only performed when the native valve was not possible to repair. In the primary evaluation using the *t*-test, a significant difference was reported between the mean values of both measurements at all the three time points (*p *< 0.001). During sub-analysis of pulmonary artery pressure to define the presence and severity of PH, both modalities show same probability of classification as reported in EuroSCORE II [6]; no PH if SPAP is between 0–30 mmHg, moderate PH if SPAP is between 31–55 mmHg, and severe PH if SPAP exceeds 55 mmHg.

The significant difference between both measurements was repeated in the sub-analysis in patients who presented with moderate or severe PH during all the three stages of assessment. Additionally, Bland-Altman analysis showed that the echocardiographic measurement underestimate the SPAP values in comparison to the PAC measurement as these two measurements are significantly different from one another and do not provide a useful level of agreement. It reported a bias between both measurements of 2.96 mmHg (95% limits of agreement –9.64 to + 15.55 mmHg) before the use of HLM, a bias of 4.80 mmHg (95% limits of agreement –10.18 to + 19.78 mmHg) after weaning from HLM and a bias of 4.23 mmHg (95% limits of agreement –7.58 to + 16.04 mmHg) at the end of surgery. The underestimation of PH in comparison to PAC warn the physicians about the clinical condition of these patient. When severe PH would be diagnosed, patients would require special perioperative (i.e., pre-, intra- and postoperative) RV support and management. This subgroup is of most importance as patients with undiagnosed severe PH could develop several postoperative complications as earlier reported [3, 4]. Hence, in patients presented with MR, Doppler echocardiography could assess the presence of pulmonary hypertension with high probability. This assessment is however underestimated and the use of PAC in those patients to diagnose, classify and monitor the therapy of PH remains recommended if required.

## 5. Limitation

Our study was performed at a single institution including a relatively small cohort of patients; however, it represents one of the first studies that investigates the difference between invasive and non-invasive SPAP measurement simultaneously in patients presenting with mitral valve regurgitation in a prospective comprehensive manner. Both approaches were performed in intubated and ventilated patients, thereby, the SPAP could have been underestimated in some patients due to anesthesia-induced vasodilation and hypotonia, besides, the fluid status, ventilation and catecholamine doses might have influenced the value of pulmonary artery pressure. Even though, due to the simultaneous measurement process we assume that these factors affects both measurement equally. The main reason for pulmonary artery pressure overestimation is the inability to identify the complete tricuspid regurgitation signal [19], so we excluded all patients without complete TR signal to avoid any overestimation of SPAP obtained by transesophageal echocardiography. Additionally the angel deviation during transesophageal echocardiography could underestimate the maximum jet velocity over the tricuspid valve. Moreover, during cardiac surgery under general anesthesia, the vasodilative effect of anesthetic medication resulted in an underestimated wedge pressure, which could be higher during normal physiological status i.e. awake patients.

## 6. Conclusions

In patients presented with mitral valve regurgitation, transesophageal Doppler echocardiography is a useful and noninvasive modality for initial measurement of pulmonary artery pressure when comparted to invasive measurement using PAC. These echocardiographic measurements however underestimate significantly the SPAP measurement in comparison to PAC. Hence, right-side heart catheterization using PAC remains precise and should be applied in patients classified with severe PH by echocardiography, whenever to specify the diagnosis, severity, and management of PH is indicated.
